# Community patterns of stigma towards persons living with HIV: A population-based latent class analysis from rural Vietnam

**DOI:** 10.1186/1471-2458-11-705

**Published:** 2011-09-18

**Authors:** Anastasia Pharris, Nguyen Phuong Hoa, Carol Tishelman, Gaetano Marrone, Nguyen Thi Kim Chuc, Ruairí Brugha, Anna Thorson

**Affiliations:** 1 Division of Global Health/IHCAR, Department of Public Health Sciences, Karolinska Institutet; Stockholm, Sweden; 2 Health Systems Research Project, Hanoi Medical University; Hanoi, Vietnam; 3 Department of Learning, Informatics, Medical Management and Ethics (LIME), Medical Management Center, Karolinska Institutet; Stockholm, Sweden; 4 Department of Epidemiology and Public Health, Population Health Sciences Division, Royal College of Surgeons in Ireland; Dublin, Ireland

**Keywords:** Vietnam, HIV, stigma, Filabavi, latent class analysis

## Abstract

**Background:**

The negative effects of stigma on persons living with HIV (PLHIV) have been documented in many settings and it is thought that stigma against PLHIV leads to more difficulties for those who need to access HIV testing, treatment and care, as well as to limited community uptake of HIV prevention and testing messages. In order to understand and prevent stigma towards PLHIV, it is important to be able to measure stigma within communities and to understand which factors are associated with higher stigma.

**Methods:**

To analyze patterns of community stigma and determinants to stigma toward PLHIV, we performed an exploratory population-based survey with 1874 randomly sampled adults within a demographic surveillance site (DSS) in rural Vietnam. Participants were interviewed regarding knowledge of HIV and attitudes towards persons living with HIV. Data were linked to socioeconomic and migration data from the DSS and latent class analysis and multinomial logistic regression were conducted to examine stigma group sub-types and factors associated with stigma group membership.

**Results:**

We found unexpectedly high and complex patterns of stigma against PLHIV in this rural setting. Women had the greatest odds of belong to the highest stigma group (OR 1.84, 95% CI 1.42-2.37), while those with more education had lower odds of highest stigma group membership (OR 0.45, 95% CI 0.32-0.62 for secondary education; OR 0.19, 95% CI 0.10-0.35 for tertiary education). Long-term migration out of the district (OR 0.61, 95% CI 0.4-0.91), feeling at-risk for HIV (OR 0.42, 95% CI 0.27-0.66), having heard of HIV from more sources (OR 0.44, 95% CI 0.3-0.66), and knowing someone with HIV (OR 0.76, 95% CI 0.58-0.99) were all associated with lower odds of highest stigma group membership. Nearly 20% of the population was highly unsure of their attitudes towards PLHIV and persons in this group had significantly lower odds of feeling at-risk for HIV (OR 0.54, 95% CI 0.33-0.90) or of knowing someone with HIV (OR 0.32, 95% CI 0.22-0.46).

**Conclusions:**

Stigma towards PLHIV is high generally, and very high in some sub-groups, in this community setting. Future stigma prevention efforts could be enhanced by analyzing community stigma sub-groups and tailoring intervention messages to community patterns of stigma.

## Background

HIV has existed in Vietnam since at least 1990, with 0.44% of the adult population estimated to be infected in 2010 [1]. As typifies a concentrated HIV epidemic, certain sub-groups of the population, such as injecting drug users (IDU), commercial sex workers (CSW), clients of CSW, and men who have sex with men, have far higher national HIV prevalence estimates than the general population (30%, 9%, 2%, and 2%, respectively) [1]. Much of the focus of HIV prevention and testing in Vietnam has been in the largest cities and the northeastern coastal provinces that are estimated to have the highest HIV prevalence. Less attention has been paid to the situation of HIV within rural areas, where more than 70% of the population of Vietnam is located, and where there is considerable employment migration to and from the higher HIV prevalence areas [2,3].

The close association between HIV infection and HIV-related risk behaviors has been emphasized by Vietnamese media and government information campaigns, where CSW and IDU are often referred to as "social evils" by the government and the wider population alike [4]. Unfortunately this alienation of risk groups has resulted in a high level of stigma being attached not only to drug use and sex work, which are heavily condemned and illegal in Vietnam, but also to persons who are known or rumored to be living with HIV [5,6]. HIV risk is equated with drug use and sex work by much of the population, who show little awareness of the growing transmission of HIV to clients of CSW or to transmission within long-term heterosexual relationships where one member of the couple engages in potentially high HIV risk behavior such as drug use, sex work, or visits to sex workers [4,7]. Stigma against members of these key populations and against persons living with HIV are very closely associated and are often referred to as "layered stigma".

Stigma against persons living with HIV has been found to be high overall and considerably higher in rural settings within Vietnam than in urban settings [8]. Also, when similar measures have been used, persons in Vietnam have been found to express more stigma against PLHIV than persons in settings with generalized HIV epidemics, where HIV epidemiology and stigma dynamics are very different, such as South Africa or Botswana [9,10]. The Government of Vietnam has recognized that stigma against PLHIV is in need of attention and in 2006 strengthened legislation and extended protection for PLHIV by promoting their rights to HIV-related confidentiality, medical care, and to integration within the community, as well as prohibiting HIV-related stigma and discrimination [11,12]. The law on HIV/AIDS is new and research has yet to be produced on its effects on stigma and discrimination towards PLHIV in Vietnam.

Existing community stigma towards PLHIV, as well as groups thought to be vulnerable to HIV such as sex workers or drug users, impacts the likelihood that those at-risk for or infected with HIV would seek preventative or care services. For PLHIV within and outside of Vietnam, fears of HIV-related stigma and discrimination or the direct experience of it have been described as leading to internal stigma, self-isolation and low self-esteem, including non-disclosure of HIV status and avoidance of contacts with support networks and health care services [13-15]. Evidence from Vietnam shows that stigmatizing attitudes towards PLHIV can act as a major barrier to adherence to HIV treatment [16] and it has been documented that persons with HIV often present very late for care, thereby reducing their opportunities for treatment success. Also for those who do not know their HIV status, stigmatizing community attitudes towards PLHIV can serve as a barrier to seeking HIV testing as well as to internalizing HIV-prevention messages, because risk reduction strategies such as condom use are associated with negative attitudes and fears about HIV [17-22]. Therefore, stigmatizing community attitudes towards PLHIV affect the quality of life and health outcomes of persons living with HIV, and could potentially result in more transmission of HIV as people delay knowing their HIV status or do not use measures to prevent HIV, such as condoms or needle and syringe programmes, in order to avoid the negative association that these have with HIV.

And so, while it has been documented that stigma against PLHIV has negative effects both on PLHIV and on efforts to prevent HIV in the wider community, interventions to significantly reduce this stigma are few [23]. Furthermore, when interventions are employed, there is a paucity of information to inform them or to measure their progress [24]. Recently, increasing effort has been made to develop and validate quantitative stigma scales or indicators for HIV-related stigma [9,25-27]. This approach brings new possibilities for measuring and comparing HIV-related stigma over time or across populations, which is important in determining whether efforts to decrease stigma towards PLHIV are working or in need of increased attention. However, much of this research has measured stigma as a total community or population score and this mean score may not give the full picture of the complex phenomena of stigma, which is often multi-faceted within individuals and populations. Only a few studies have evaluated the determinants to HIV-related stigma [9,22,28], and these have done so using a variable-centered approach.

Another analysis approach that could enhance understanding of the varying character and levels of HIV-related stigma within a population over time is latent class analysis. Latent class analysis is a "respondent-centered" approach that seeks to group individuals into class groups based on their responses to a set of observed variables (in this case, responses to eight stigma statements which relate to stigma against PLHIV)[29]. Latent class analysis has been used widely in market research to tailor marketing campaigns to segments of the population as well as to understand patterns of complex health risk behaviors [30], including substance abuse [31], mental health [32,33], as well as HIV/AIDS risk behavior, knowledge and programming [10,32,34-36]. In addition to understanding the pattern of stigmatizing attitudes present in each class by examining the probability of each answer to the stigma statements on which the classes were composed, factors independently associated with the stigma class membership can be identified through latent class regression [29]. In the present study, we explore whether latent class analysis can be used to analyze the patterns of HIV-related stigma towards PLHIV and to identify predictors of different levels of stigma in a population-based rural sample.

## Methods

### Study setting

This study was conducted in the rural farming district of Bavi, Ha Tay province, located 60 kilometers to the northwest of Hanoi with a population of about 262,000. HIV prevention messages in the area, and throughout rural Vietnam, are generally disseminated through mass media and billboards at district and communal health stations, and through local government or women's unions. In 2007, the reported notification rate of HIV in Bavi district was 0.12%. Since more than 92% of the population has never tested for HIV, including nearly 80% of those who report feeling at-risk for HIV, and because those cases detected are often detected at a very advanced stage of illness, it is likely that a significant proportion of those living with HIV are not aware of their HIV status and that prevalence is higher than the notification rate [37], (Personal communication, Bavi District Preventative Health Director, 25^th ^Sept 2008).

### Study population

This study was conducted within a rural demographic surveillance site (DSS), Filabavi, during April-May 2007. Filabavi DSS began in 1999 in Bavi district, Ha Tay province (Ha Tay became part of Hanoi province in 2008). The district was divided into 352 geographic clusters that were then stratified into four geographic regions (highland, lowland, mountainous and island). Seventy-one of these were randomly sampled with a probability of inclusion proportional to cluster population size to make up the DSS sample. Quarterly surveys of vital events have been carried out within Filabavi DSS on the entire sample of 12,818 households including 50,456 individuals (2007 population numbers) since 1999. For the present cross-sectional survey, a two-stage cluster sampling method was used to, first, randomly sample 46 of the 71 clusters and to, then, randomly sample 1874 adults (18-60 years) stratified by age and sex from the adult sample within each cluster. The mean age for men and women in the study sample was 37.4 years; additional socio-demographic characteristics of the study sample are shown in Table 1.

**Table 1:** Socio-demographic characteristics of the study sample

	Total sample		
	**Women** **n = 943** **n (%)**	**Men** **n = 931** **n (%)**	**Total** **n = 1874** **n (%)**

**Age**			
18-29 years	313 (33)	304 (33)	617 (33)
30-44 years	321 (34)	314 (34)	635 (34)
45-60 years	309 (33)	313 (34)	622 (33)
**Education level**			
Primary (≤ 6 years)	179 (19)	161 (17)	340 (18)
Secondary (7-12 yrs)	676 (72)	664 (71)	1340 (71)
Tertiary	88 (9)	106 (11)	194 (10)
**Economic status**			
Poorest 60%	574 (61)	557 (60)	1131 (60)
Least poor 40%	369 (39)	374 (40)	743 (40)
**Place of residence**			
Lowland	178 (19)	167 (18)	345 (18)
Highland	492 (52)	491 (53)	983 (52)
Mountainous	250 (27)	252 (27)	502 (27)
Island	22 (2)	21 (2)	43 (2)
**Ethnic group**			
Kinh	898 (95)	895 (96)	1793 (96)
Non-Kinh	45 (5)	36 (4)	81 (4)
**Long-term out-migration**	84 (9)	149 (16)	233 (12)
**Heard of HIV from > 3 sources**	174 (18)	217 (23)	391 (21)
**Feels at risk for HIV**	90 (10)	131 (14)	221 (12)
**Knows someone with HIV**	389 (41)	376 (40)	765 (41)

Notes: Due to rounding, some percentages may not total 100%. Economic status was missing for 20 individuals and information on place of residence and out-migration was missing for one individual.

### Ethics

Research ethics permission was sought and granted from Hanoi Medical University, Hanoi, Vietnam. Individuals were included in the study after the study purpose had been explained and verbal informed consent given.

### Data collection

A study-specific questionnaire was developed based on the concepts of stigma presented by Link and Phelan (2001) of labeling, stereotyping, separation, status loss and discrimination--all items which were theorized to lead PLHIV to be less open about their HIV status and to inhibit them from seeking preventative, testing and/or treatment services. We also theorized that these items would be associated with less openness toward HIV preventative information and knowledge among those not knowingly living with HIV. The items included have also been used in common stigma scales [9,25,38]; and were congruent with formative qualitative research that we conducted in this area with persons living with HIV (unpublished data). From these, eight statements about persons living with HIV were asked with possible responses, "Yes", "No", or "Not sure/maybe". The questionnaire was pilot-tested and we assessed through "think aloud" exercises [39] that persons who responded that they were "not sure" or "maybe" to a statement had actually understood the question. Minor revisions in the questionnaire were made prior to data collection. Female surveyors who received study-specific training used structured questionnaires to interview participants in a private area inside of or nearby the participants' homes. Interviews took place during routine quarterly DSS data collection. As part of the DSS, data collection is supervised and data quality checked by six trained supervisors and a field coordinator.

### Data analysis

Data were entered in EpiData version 3.1 (Odense, Denmark). STATA version 9.0 (College Station, Texas, USA) and the poLCA package [40] for the open-source software R version 2.10.1 (R Development Core Team, 2009) were used for data processing and analysis. Household socioeconomic and migration data collected during regular DSS rounds since 1999 were linked to the individuals in this survey.

A dichotomous variable for long-term outmigration was calculated for having left (and returned to) the district for greater than three months at least one time during the 7 years prior to the study (see Table 1). Another dichotomous variable for "heard of HIV from more sources" was created for spontaneously listing 3 or more sources from which the respondent had heard of HIV. Economic status was calculated based on principal components analysis of household assets and dichotomized into the top 40% and bottom 60% of the study sample [41].

Descriptive data analysis was conducted first in order to understand frequencies of the socio-demographic variables, HIV risk variables, and the stigma statements. Then, Pearson's Chi-square test was employed to investigate statistically significant associations between the individual HIV stigma statements and relevant socio-demographic and HIV risk variables. Thereafter, using the set of categorical responses ("Yes", "Not sure/maybe", or "No") to the eight stigma statements, latent class analysis was performed to organize respondents into meaningful groups based on the stigmatizing attitudes that they expressed about persons living with HIV.

We determined an underlying latent class structure for HIV-related stigma, using the criteria of minimizing the values for Akaike Information Criteria (AIC) and Bayesian Information Criteria (BIC) combined with practical and theoretical usefulness of the final class structure [29]. Missing values for any of the manifest variables were removed from the analysis, resulting in a total sample size of 1764 persons that was used for the latent class analysis. In our analysis, (see Table 2) the AIC and BIC values for the three- and four-class solutions were very similar and after examining both solutions, we chose the three-class solution as it was determined to be more practically and theoretically useful for public health practice. We then evaluated correlates of latent class membership with latent class regression. A multinomial logistic regression model was constructed to identify factors independently associated with the dependent variable: stigma group (or class) membership. Lowest stigma group was chosen as the reference group. Independent variables significant in bivariate analysis with at least one of the eight stigma statements at a level of p < 0.10 were included in the model using a stepwise forward selection procedure. Pairwise interaction was evaluated between variables in the final model and no significant interaction was found. The best regression model was assessed by minimizing the Chi-square goodness of fit [40]. Odds ratios with 95% confidence intervals were computed. A value of p < 0.05 was considered statistically significant in the final model.

**Table 2:** Fit indices for latent class analysis of stigmatizing attitudes towards PLHIV

	AIC	BIC
**2 classes**	23853	23077
**3 classes**	22388	22749
**4 classes**	22121	22618
**5 classes**	24368	25002

AIC: Akaike Information Criteria;

BIC: Bayesian Information Criteria

## Results

### Overall stigma

Results indicate very high stigma towards PLHIV among both men and women of all ages in this rural population. More than half of respondents agreed that PLHIV were promiscuous (67%), should feel ashamed (64%), and conjectured that they themselves would feel ashamed if a family member had HIV (69%) (Table 3). About half of the respondents agreed that PLHIV should be isolated. Attitudes about interacting with PLHIV in common situations of interpersonal contact varied, with over half of the respondents stating that they would not want to be friends with someone with HIV (60%) and that it was not safe for a child to play with someone with HIV (70%). Fewer (44%) respondents stated that they would not share a meal with someone with HIV and about 17% responded that an HIV-positive student who was not sick should not be allowed to continue school. A significantly greater proportion of women than men held stigmatizing attitudes on seven of the eight stigma-related statements (Table 3). More than 90% of all respondents of both sexes agreed with two or more stigmatizing statements about persons living with HIV (data not shown).

**Table 3:** Attitudes towards PLHIV among Vietnamese rural adults

	Yes n (%)	No n (%)	Don't know/Not sure n (%)
1. PLHIV should be isolated* n = 1835	922 (50)	768 (42)	145 (8)
2. I would be ashamed if someone in my family had HIV* n = 1829	1259 (69)	178 (10)	392 (21)
3. PLHIV are promiscuous n = 1832	1232 (67)	140 (8)	460 (25)
4. PLHIV should feel ashamed* n = 1823	1174 (64)	327 (18)	322 (18)
5. I would like to be friends with someone with HIV** n = 1819	410 (22)	1084 (60)	325 (18)
6. I would share a meal with someone with HIV** n = 1832	776 (42)	801 (44)	225 (14)
7. It is safe for children to play with PLHIV* n = 1834	342 (19)	1279 (70)	213 (12)
8. A student with HIV who is not sick should be allowed to continue school* n = 1830	1160 (63)	315 (17)	355 (19)

Notes: Percentages may not total 100 due to rounding to the nearest integer. The total sample for each statement varies due to non-response to some statements by some participants. Statements 1-4 are phrased so that answering 'Yes' indicates a more stigmatizing attitude and statements 5-8 are phrased so that 'No' indicates a more stigmatizing attitude. The questions were not asked in this order, but are grouped in their presentation for clarity.

*Significant difference at p < .05 for women reporting a more stigmatizing attitude than men

**Significant difference at p < .001 for women reporting a more stigmatizing attitude than men

### Population distribution of stigma against PLHIV

The latent class analysis generated three classes of stigma ranging from lowest to highest degree of expressions of stigmatizing attitudes towards PLHIV (Table 4). Within these three class groups, the first, comprised of about 43% of the population, included persons who had the lowest probability of expressing a stigmatizing statement (therefore called *least stigmatizing)*. However, even in the *least stigmatizing *group, expressions of stigma were fairly high, particularly for statements that PLHIV should feel ashamed or that one would feel ashamed if a family member had HIV, that PLHIV are promiscuous, and that it is not safe for a child to play with a PLHIV. The second class consisted of about 19% of the study population and included persons who were likely to express strong stigmatizing views on some but not all of the statements or who were most likely to state that they were unsure or did not know in response to the statement. This class is referred to as *ambivalent*. The third class, comprised of about 38% of the population, included persons who had the highest probability of a stigmatizing response on most of the eight statements (*highly stigmatizing*).

**Table 4:** Basic class structure: Three latent-class model of stigmatizing attitudes towards PLHIV, (n = 1764)

	Latent class								
Assigned label	**1** ***Less stigmatizing***			**2** ***Ambivalent***			**3** ***Highly stigmatizing ***		

*Probability of membership*	0.43			0.19			0.38		

Conditional probability of a stigmatizing response*									

	**1** least stigma	**2** not sure/ don't know	**3** most stigma	**1** least stigma	**2** not sure/ don't know	**3** most stigma	**1** least stigma	**2** not sure/ don't know	**3** most stigma

1. PLHIV should be isolated	**0.74**	0.05	0.21	0.16	0.24	**0.60**	0.19	0.02	**0.79**

2. I would be ashamed if someone in my family had HIV	0.21	0.19	**0.60**	< 0.01	**0.49**	**0.51**	0.02	0.07	**0.90**

3. PLHIV are promiscuous	0.13	0.23	**0.64**	0.02	**0.46**	**0.53**	0.05	0.16	**0.79**

4. PLHIV should feel ashamed	0.32	0.17	**0.51**	0.02	**0.43**	**0.55**	0.09	0.05	**0.85**

5. I would like to be friends with someone with HIV	**0.51**	0.21	0.28	< 0.01	**0.38**	**0.62**	0.01	0.03	**0.95**

6. I would share a meal with someone with HIV	**0.81**	0.07	0.13	0.22	**0.49**	0.29	0.08	0.04	**0.88**

7. It is safe for children to play with PLHIV	0.35	0.15	**0.49**	0.03	0.18	**0.79**	0.08	0.03	**0.89**

8. A student with HIV who is not sick should be allowed to continue school	**0.91**	0.04	0.05	**0.46**	**0.44**	0.09	0.40	0.24	0.36

* Recoded from original yes or no answers so that the least stigmatizing response is 1 and the most stigmatizing response is 3.

Note: **Bolding **denotes the most frequent responses within the class to each statement. Responses may not total 1.0 due to rounding.

### Factors associated with stigma group membership

The results of the multinomial logistic regression for *highly stigmatizing *and *ambivalent *groups, with *least stigmatizing *as the reference group, are presented in Table 5. Female sex was found to be significantly associated with most stigmatizing attitudes against persons living with HIV (i.e., belonging to the *highly stigmatizing *group) (OR 1.84, 95% CI 1.42-2.37). However, some factors seemed to be protective against *highly stigmatizing *group membership including: greater educational attainment (OR 0.45, 95% CI 0.32-0.62 for secondary education; OR 0.19, 95% CI 0.10-0.35 for tertiary education), long-term migration out of the district (OR 0.61, 95% CI 0.4-0.91), feeling at-risk for HIV (OR 0.42, 95% CI 0.27-0.66), having heard of HIV from more sources (OR 0.44, 95% CI 0.3-0.66), and knowing someone with HIV (OR 0.76, 95% CI 0.58-0.99) (Table 5). While sex, educational attainment and migration were not significantly associated with *ambivalent *class membership, those who heard about HIV from more sources (OR 2.01, 95% CI 1.41-2.88) had greater odds of belonging to the *ambivalent *group. Those who felt at-risk for HIV (OR 0.54, 95% CI 0.33-0.9) or who reported knowing someone with HIV (OR 0.32, 95% CI 0.22-0.46) had lower odds of belonging to the *ambivalent *group than to the *least stigmatizing *group.

**Table 5:** Risk predictors for stigmatizing attitudes towards persons living with HIV among rural Vietnamese adults (n = 1764)

*Reference group is Class 1 (least stigmatizing)*	Class 2 Ambivalent aOR (95% CI)	Class 3 Highly stigmatizing aOR (95% CI)
Sex		
Male	1.0	1.0
Female	1.09 (0.79-1.50)	1.84 (1.42-2.37)**

Long-term outmigration		
No	1.0	1.0
Yes	0.84 (0.53-1.33)	0.61 (0.40-0.91)*

Education		
Primary	1.00	1.00
Secondary	0.78 (0.48-1.27)	0.45 (0.32-0.62)**
Tertiary	1.12 (0.60-2.09)	0.19 (0.10-0.35)**

Heard of HIV from more sources		
No	1.0	1.0
Yes	2.01 (1.41-2.88)**	0.44 (0.30-0.66)**

Feels at risk for HIV		
No	1.0	1.0
Yes	0.54 (0.33-0.90)*	0.42 (0.27-0.66)**

Knows someone with HIV		
No	1.0	1.0
Yes	0.32 (0.22-0.46)**	0.76 (0.58-0.99)*

Notes: aOR = adjusted odds ratio; data are reported as aORs adjusted by all other variables in the model.

* p < 0.05 ** p < 0.001

## Discussion

Our study is the first in Vietnam that quantitatively examines the patterns of stigmatizing attitudes towards PLHIV within a rural population-based sample with latent class analysis. We found that women and individuals with less formal education, in particular, held the most highly stigmatizing attitudes. There was also evidence that those who had heard of HIV from fewer sources, who did not know anyone with HIV, and whom did not feel at-risk for HIV reported the most stigmatizing attitudes towards PLHIV.

Three discrete groups emerged based on similar within-group responses to statements about PLHIV. Those who held the most stigmatizing attitudes towards PLHIV appeared to differ from those with least stigmatizing attitudes in that they were more likely to be women, to have less education, and not to have migrated out of the area. The inverse relationship between years of education and level of HIV-related stigma was reported in South Africa [9]. Hong et al [42], qualitatively describe high HIV-related stigma among Chinese rural-to-urban migrants, and it is likely that those who have not migrated outside of rural areas in our study sample had less likelihood of being exposed to HIV information and stigma reduction campaigns which target urban areas. The relationship between gender and HIV-related stigma is less well-documented, and might be more unique to the Vietnamese context, where women are socially expected to distance themselves from behaviors considered to be "social evils" such as drug use and sex work [4,42].

We also saw that those who had heard of HIV from fewer sources were significantly more likely to belong to the *highly stigmatizing *group, perhaps indicating that persons in this group had been exposed to or remembered less HIV information. However, the role of media and information campaigns was complex, as those who had heard of HIV from more sources had about twice the odds of belonging to the *ambivalent *than the *least stigmatizing *group, perhaps indicating that persons in the *ambivalent *group had heard of and remembered HIV information sources that were conflicting, confusing or ambiguous in their messages about HIV and PLHIV. Vietnam's National Action Plan for HIV states that HIV stigma reduction campaigns are to be carried out through the media, schools, workplaces and with the help of famous personalities [6]. Critiques of government-driven information campaigns point to how they closely link HIV to the "social evils" of CSW and IDU, and the evidence from this study does not refute the presence of that link in community attitudes [4]. Especially among those who were *ambivalent *in regards to their attitudes towards PLHIV, further analysis of these information campaigns is necessary to assess the extent to which these are effective or, instead, inadvertently sustain HIV-related stigma by associating infection with "social evils" or by providing incomplete or ambiguous HIV prevention messages [4].

Those who reported knowing someone with HIV or feeling personally at-risk for HIV were significantly more likely to belong to the *least stigmatizing *group. This has been found in Asian settings and elsewhere and points to the potential of putting a "human face" on HIV as a stigma-reduction or a stigma-prevention measure [9,25,43]. However, in a concentrated HIV epidemic setting, such as Vietnam, where HIV prevalence is low, the likelihood of knowing someone with HIV will also be low as compared to the likelihood in a country with a generalized HIV epidemic. We did not assess what it meant to "know" someone with HIV in our study and have not seen this assessed elsewhere. However, these results should be taken into account when assessing the stigma-reducing potential of programs in which PLHIV "go public" and provide community education. In addition to providing HIV prevention information, this could have the potential to reduce HIV-related stigma based on the "human face" associated with HIV [44].

Our study generally found similar community stigma towards PLHIV as compared to studies conducted in 2005 in other regions of Vietnam [8,45] and higher HIV-related stigma than reported outside of Vietnam [9,10]. For example, in Southern Vietnam, Nguyen Anh Tuan, et al found that the same percentage of persons in rural Southern Vietnam as in our study (64%) believed that PLHIV should feel ashamed while the urban sample from the same study reported lower stigma (45%) [8]. Reports on the measure "PLHIV should feel ashamed" from non-Vietnamese settings are much lower ranging from 8-34% in various South African settings [9]. Fewer persons in Botswana report that they would be reluctant to share a meal with a PLHIV (27%) as compared to 44% in our sample [10]. There is some evidence of a relationship between the availability of treatment for HIV (ART) and lower attitudes of stigma [10,28]. This, in combination with the greater likelihood of knowing someone with HIV, could explain the lower levels of stigma in urban Vietnam, where ART became available in 2004, as compared to this rural district where it was still unavailable in 2007.

In addition to quantitatively describing patterns of stigma within a population, latent class analysis can provide important information for HIV stigma-reduction interventions within communities. A latent class analysis can be used in conjunction with an "audience segmentation" approach [46], a key concept in commercial and social marketing, to divide the population into sub-groups. Then, intervention strategies can be tailored to the sub-groups' demographic characteristics, knowledge levels and, in the case of stigma against PLHIV, into groups with similar stigma attitudes. In this community, for example, interventions would likely need to approach men and women differently, as gender was an important variable which differentiated the stigma groups. Other factors, such as differences in stigma patterns based on knowing a person with HIV or feeling at personal risk for HIV might also direct intervention and health promotion strategies for this community. It is also of interest that almost 20% of the population was *ambivalent *with regard to many of the stigma statements and that these persons appeared to have heard of HIV from more sources. Persons in the *ambivalent *group may have the potential to be influenced towards more accepting attitudes and this could be evaluated by applying targeted, high-quality anti-stigma messages and by following-up over time to determine whether the intervention had a sustained impact. Finally, in addition to informing the stigma-reduction needs within the community, latent class analysis could also be used as an evaluation technique to measure community patterns of stigma over time so that changes in group composition or number can be evaluated. This approach might be superior to overall community stigma scores followed over time, which give less detailed information on the distribution of people across stigma classes.

### Methodological considerations

Of note, in pilot testing and in subsequent data collection, a sizable portion of the sample responded "Not sure/maybe" to each of the stigma statements (range 8%-25% of respondents, depending on the question). We checked and understanding of the questions was consistently high. Respondents explained that they were ambivalent because they wished to qualify their response based on how sick the PLHIV was or how the person had become infected with HIV. We took the view that these responses were important and typified the reluctance that some community members often have in determining their attitudes towards persons living with HIV and, therefore, kept the "Not sure/maybe" response as a separate category for data collection and analysis. This is an important consideration for those trying to measure stigma in the Vietnamese context and may be an important measure to assess qualitatively and while validating stigma scales in other settings as well.

While our study has the strength of using population-based data, it might be limited due to social desirability bias. However, respondents expressed higher-than-expected stigma against PLHIV and interviews were conducted by well-trained interviewers in a private area to minimize social desirability bias. Women in this study reported more highly stigmatizing attitudes than men and this might have been an effect of the study's use of only female interviewers. If stigma were easier to express to someone of the same gender, then the degree of stigma toward PLHIV among men could have been under-estimated. Since this study was exploratory in nature, we used commonly used stigma items that, in qualitative research that we had conducted, had been linked to less uptake of HIV prevention, testing and care services. Future research could benefit from the use of a validated stigma scale, which can be repeated over time and compared across settings. Finally, often in the analysis of data where cluster sampling is used, such as in this study, the design effect or some other manner of taking sample weights into account is performed. It was not possible to take this into account using R software, nor using other software that were available to us. This may have led to somewhat biased estimates as compared with what would have been achieved if we had used a simple random sample. However, since the number of clusters was large (42 of the total 71) and cluster of origin did not appear to significantly differ with respect to answers on the eight stigma statements in descriptive analysis, we believe that this bias does not seriously influence the study findings.

## Conclusions

Stigma against persons living with HIV remains a significant issue in concentrated epidemic settings such as Vietnam. Despite the existing legal framework prohibiting discrimination and the focus of policy and government education programs on stigma reduction for more than a decade, stigma against persons living with HIV appears to be widespread and could present an obstacle to the individual and community uptake of HIV prevention messages as well as a barrier to care for PLHIV in need of testing, treatment and support. Attention must be given to the nature and quality of HIV stigma-reduction, focusing on strategies and unambiguous anti-stigma messages that are tailored to specific stigma sub-group characteristics. Analysis of community-specific patterns of stigma using latent class analysis in order to tailor anti-stigma interventions according to community class characteristics is one approach that could lead to greater understanding of how to target and track community interventions to reduce stigma against persons living with HIV.

## Competing interests

The authors declare that they have no competing interests.

## Authors' contributions

AP, AT, NTKC, and NPH conceived of the study and participated in its design. AP, NPH and NTKC coordinated data collection. AP and GM conducted data analysis. AP completed the first draft of the manuscript, and all authors contributed to commenting on the analysis and manuscript. All authors read and approved the final manuscript.

## Pre-publication history

The pre-publication history for this paper can be accessed here:

http://www.biomedcentral.com/1471-2458/11/705/prepub
